# Weldability Improvement of Additively Manufactured Nickel-Based Superalloy by Controlling W and Si Migration at Solidification Front

**DOI:** 10.3390/ma19153210

**Published:** 2026-07-27

**Authors:** Rui Ma, Xin Xi, Zhaoyang Lin, Wenrui Luo, Yanhao Hou, Wenjun Zhao, Zhifeng Shi, Xiaoguo Song, Qiang Chen, Danyang Lin

**Affiliations:** 1 Beijing Power Machinery Institute, Beijing 100074, China; 2 Academy of Aerospace Liquid Propulsion Technology, Xi’an 710100, China; 3 National Key Laboratory of Precision Welding & Joining of Materials and Structures, Harbin Institute of Technology, Harbin 150001, China; 4 Ningbo Zhongyuan Advanced Materials Technologies Co., Ltd., Ningbo 315000, China

**Keywords:** weldability, additively manufactured superalloy, hot cracking, elemental migration regulation

## Abstract

Nickel-based superalloy components prepared by laser powder bed fusion (LPBF) are increasingly being used in the hot ends of aeroengines, which still need to be laser-welded to realize the assembly connection of large closed structures. However, the unique microstructural characteristics of LPBFed parts render them more poorly weldable than casts and wroughts, especially in cracking-sensitive superalloys. In this study, cracking mechanisms were analyzed by performing laser deep fusion welding on an LPBFed Haynes 230 alloy. The key factors leading to weld cracking were strongly correlated with the low-melting-point TCP phases enriched with W, Si, Al, and silicides, both of which tended to be distributed in the boron(B)-rich region and exhibited poor coherence with the matrix. In addition, the flow behavior of the molten pool affected the solidification rate of the weld, resulting in large solidification shrinkage stresses in the cracking-sensitive zone (CZ) part of the weld. In order to minimize the development conditions of the cracking-sensitive phases, reducing the content of W (from 14.96 to 13.56 wt.%) and Si (from 0.47 to 0.25 wt.%) was chosen to alleviate the segregation of W, Si and C elements in the solid phase at the end of solidification. Thus, TCP phase and silicides were transformed into carbides, which successfully suppressed weld cracking, reducing the crack depth ratio from 0.70 ± 0.08 to zero. New insights into the weldability improvement and crack inhibition mechanism of laser-welded additively manufactured cracking-sensitive superalloys are provided, accelerating the rapid application of large assemblies.

## 1. Introduction

The Haynes 230 superalloy features excellent strength, high-temperature resistance, oxidation resistance, creep resistance, etc., and plays an essential role in the hot-end components of aerospace engines [1,2,3]. The aerodynamic shapes and flow path designs of engine parts for high-temperature resistance enable the widespread use of laser powder bed fusion (LPBF) technology to mold complex structures [4]. However, to prepare closed structures and large parts, reliable joining of additively manufactured components is required in combination with welding techniques [5,6,7]. Laser welding is widely used in joining aircraft parts because of its high precision, small heat-affected zone, and high welding efficiency [8,9]. However, due to their higher alloying degree, superalloys are prone to producing low-melting-point compounds or eutectics during the non-equilibrium solidification process of laser welding, leading to cracking at the weld grain boundaries (GBs) [10,11].

The content of the elements prone to segregation at the GBs and the form of the compounds determine the low-melting-point phase morphology at the GBs and the interfacial residual strain of the alloy. Yang et al. [12] clarified that the enrichment of Al, Si, and P to form low-melting-point phases leads to solidification cracking of laser-welded GH3539 alloy joints, whereas the uncracked GB locations are mainly carbides of Cr. Chen et al. [13] found that during the welding solidification stage of the Ni-28W-6Cr alloy, a higher content of Si at the end of solidification tended to form a liquid film of W, Cr, and Mn with a lower melting point, weakening the bonding of the GBs. In addition, numerous studies have found that B segregation at GBs increases the wettability of GBs and the stability of liquid films between solidified dendrites, reducing the solid/liquid surface energy and significantly increasing the susceptibility to solidification cracking [14,15]. Hu et al. [16,17] found that during the solidification of LPBFed Ni-Cr-W alloy, GB-enriched B and Si formed low-melting-point borides and Laves + γ eutectic phases with Cr and W, respectively, leading to an increased tendency of GB cracking, which was also found in other nickel-based alloys [18,19].

Compared with cast and wrought, additively manufactured (AM) parts present more severe weld crack-sensitivity due to their strong texture, high-density dislocations and uneven residual stress [20]. While extensive studies exist for weldable AM superalloys like IN 625 [5,21,22] and IN 718 [6,23], a major research gap persists regarding crack-sensitive AM superalloys containing high fractions of refractory elements, where non-equilibrium solidification conditions aggravate elemental segregation [24,25,26,27]. Although conventionally manufactured Haynes 230 alloy presents good weldability, severe weld solidification cracking occurred in the LPBFed Haynes 230 alloy [28,29]. This was attributed to the formation of a low-melting-point topologically dense phase (TCP), silicides, and borides by the segregation of W, Si, and B at the GBs, exhibiting a more sensitive cracking tendency of the AM part as well as alloy element effects [5,28]. Therefore, regulation of low-melting-point compounds at the end of solidification is crucial for inhibiting GB cracking in superalloys. To mitigate the segregation of low-melting-point elements, Ernst et al. [15] reduced weld cracks in the Haynes 230 alloy by decreasing the B content. Tomus et al. [30,31] also suppressed the alloy’s cracking by lowering the TCP phase’s volume by reducing the Si content in the GB. Therefore, reducing the content of low-melting-point phase-forming elements is also a key modifier for reducing the hot cracking tendency. In addition, the addition of rare-earth elements such as Sc, Zr, and Hf to superalloys can promote grain refinement and local microalloying at the GBs, thus inhibiting the formation of low-melting-point phases when applied in trace quantities [32,33,34]. However, under rapid non-equilibrium welding cooling rates, excessive concentrations of Zr and Hf efficiently react with C to form low-melting-point phases such as ZrC and Hf/Ti/Al/C eutectics, which expands the brittle temperature range and exacerbates the weld cracking tendency [35,36,37,38]. In summary, increasing the melting point of the GB precipitation phase and relieving the residual strain can effectively inhibit solidification cracking. Thus, in addition to controlling the B element, which is prone to forming low-melting-point compounds, suppressing the enrichment of W and Si elements is also key to hindering the cracking of Ni-Cr-W-based alloys.

Because of the difficulty in regulating the extremely low B content, this study compared the effects of alloy composition on weld cracking and GB precipitation phases by regulating the content of the low-melting-point compound-forming elements W and Si. In addition, flow field simulations and thermal dynamics (TD) calculations were combined to analyze the weld solidification behavior in cracking-prone zones. Finally, the mechanisms of weld cracking inhibition and precipitate transition were revealed. New crack-suppression insight was provided to facilitate the reliable application of laser-welded AM superalloy parts.

## 2. Materials and Methods

### 2.1. Materials and Manufacturing Processes

Three Haynes 230 pre-alloyed powders were produced by gas atomization with a nearly spherical shape and a size of 15~53 μm, named 230-A, 230-B, and 230-C alloys. Their chemical compositions are presented in Table 1. The compositions of Haynes 230 casting were measured using inductively coupled plasma atomic emission spectroscopy (ICP-AES) and are shown in Table 1, which were similar to those of 230-A. For each alloy variant, a sample size of 3 independent specimen blocks was prepared to ensure reproducibility.

Relative specimens were fabricated using an BLT-S210 laser powder bed fusion system (Xi’an BLT Additive Manufacturing Co., Ltd., Xi’an, China). Based on our previous studies, hot isostatic pressing at 1180 °C and subsequent solid solution treatment at 1180 °C were performed on the as-built specimens [28]. After LPBF and heat treatment, workpieces with dimensions of 100 mm × 100 mm × 6 mm were laser-welded using an IPG fiber laser (IPG YLS-6000) (IPG Photonics Corp., Marlborough, MA, USA) with a maximum output power of 6000 W and a spot of 1.2 mm. During the laser-welding process, a Kuka six-axis robot (KUKA AG, Augsburg, Germany) was used to control the movement of the welding head, and the welding direction (WD) was parallel to the LPBF building direction (BD). Moreover, argon-protecting gas with a flow rate of 20 L/min was employed to prevent oxidation of the molten and weld seams. Five welding process parameters were selected to evaluate the weldability across a systematic gradient of line energy densities (50.0 to 175.0 J/mm), transitioning from conduction mode to deep penetration keyhole mode to capture distinct molten pool cracking behaviors, as listed in Table 2. For each process parameter set, 5 independent experimental weld repetitions were performed to guarantee statistical representation.

### 2.2. Microstructure Analysis Method

The cross-sectional cutting of the weld seam was performed using an electric discharge machine (Wire-EDM, Suzhou Sanguang Science & Technology Co., Ltd., Suzhou, China). After sandpaper grinding and mechanical polishing, the metallographic samples were observed under an optical microscope (OM, Tokyo, Japan). A scanning electron microscope (SEM, Zeiss, MERLIN Compact, Oberkochen, Germany) with an electron probe X-ray microanalyzer was used to characterize the microstructure and elemental distribution. The OM and SEM samples were etched with 20 g of CuCl_2_, 100 mL of hydrochloric acid, and 100 mL of alcohol for 60 s. Transmission electron microscopy (TEM, FEI, Talos F200S G2) (Thermo Fisher Scientific, Waltham, MA, USA) was conducted to further analyze the nanostructure and precipitated phases on both sides of the cracks and GBs. Quantitative grain size measurements, misorientation evaluations, and chemical point-scans were calculated based on a minimum of 5 independent fields of view for each condition to generate mean statistical values and standard deviations.

### 2.3. Finite Element Simulation

A keyhole-mode three-dimensional heat transfer and fluid flow model was used to calculate the temperature gradients, cooling rates, and solidification behaviors under various welding conditions. The model considered the energy, momentum, and mass conservation equations to describe the heat transfer, melt flow behavior, and mass transfer. A volume-of-fluid algorithm is used to track the free surface of the keyhole. The laser heat sources, thermal convection, and radiation on the free surface were added to the energy term. Considering the multiple reflections of laser energy rays in the keyhole, we adopted a Gaussian distribution-like heat source model for the laser energy. The momentum conservation equation was supplemented with a series of driving forces, including the recoil pressure, buoyancy, and surface tension, the details of which are available in our previous study. The thermophysical parameters of the Haynes 230 alloy used in the heat transfer and fluid flow models were extracted using JmatPro V13.1 software. To allow for traceability of the model, the following assumptions are proposed:

(1) The shielding gas and laser-induced plasma influences are neglected.
(2) The weld pool is an incompressible Newtonian fluid that exhibits a laminar flow.

**Figure 1:**
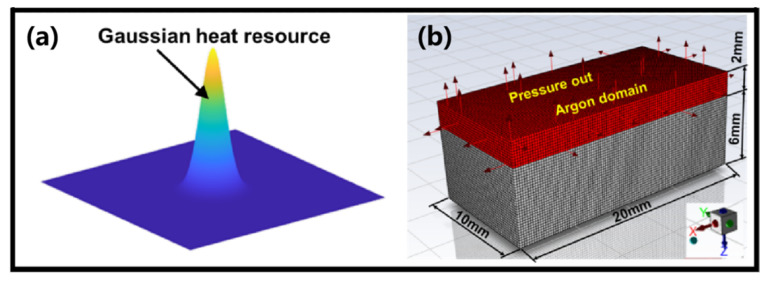
(**a**) A Gaussian heat source model; (**b**) a finite element simulation model.

Figure 1b shows the geometric model’s computational domain (20 mm × 10 mm × 8 mm) and boundary conditions. The heights of the gas and liquid domains were 2 and 6 mm, respectively. The solution domain was divided into 200,000 uniform cells with dimensions of 0.2 mm × 0.2 mm × 0.2 mm. The top and side surfaces of the gas domain were set as press-out surfaces, the bottom and side surfaces of the liquid domain were set as wall surfaces, and the wall boundary conditions, including thermal convection and radiation, were considered. Initially, the upper gas domain was filled with Ar at room temperature and ambient pressure.

## 3. Results

### 3.1. Weld Macroscopic Morphology

**Figure 2:**
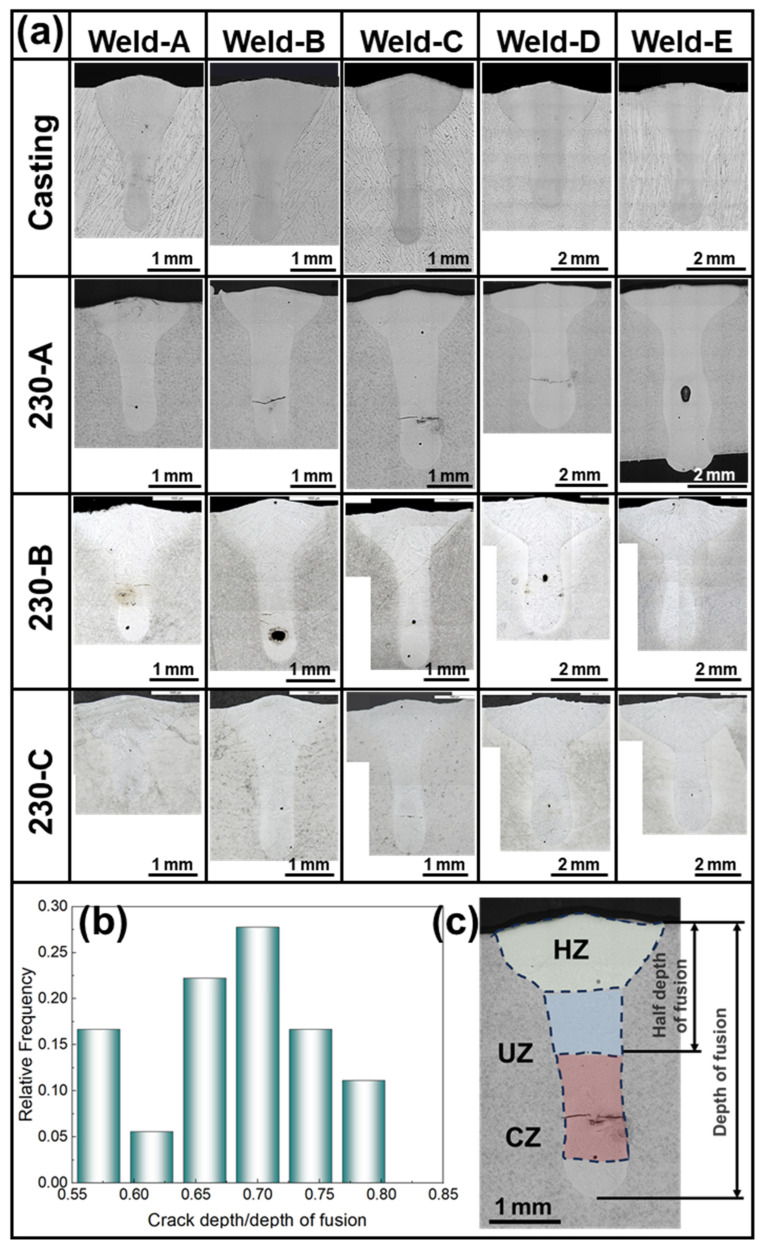
Optical morphology of three weld joints with different welding process parameters: (**a**) weld morphologies in castings and LPBF parts; (**b**) crack depth distribution for 50 weld cross-sections in LPBF parts; (**c**) schematic diagram of the HZ, UZ and CZ of the weld.

Figure 2 presents the cross-sectional optical morphology of the laser-welded joints of the Haynes 230 alloy produced by cast and LPBF. Although castings present different grain morphologies and growth directions, the welds in castings are well formed and free of cracks, suggesting great weldability, whereas the welds in the LPBF parts show severe transverse penetration cracks, indicating that the LPBF parts feature a more severe cracking susceptibility and unique cracking mechanism [39].

As shown in Figure 2a, welds on all LPBF parts consist of a trapezoidal heat-conducting zone at the top (HZ), an uncracked zone in the upper center (UZ), and a cracking-sensitive zone in the lower center of the deep melt zone (CZ). The crack depth (*d*_c_) and depth of fusion (*d*_f_) were determined for 50 weld cross-sections (Figure 2b). The *d*_c_ was mainly concentrated between 0.5 and 0.8 times the *d*_f_, so this area was set as the CZ part, while the deep melting zone above the CZ was the UZ part, as shown in Figure 2c. The macrostructural cracking susceptibility shows a direct correlation with the initial chemical compositions compiled in Table 1. Alloy 230-A exhibited significant transverse penetration cracking in the weld-A–D specimens and poor weldability. Alloy 230-B exhibited shorter transverse cracks in Weld-A–D specimens with improved weldability. In contrast, alloy 230-C cracked only in the Weld-C welding process, significantly reducing porosity and improving weldability. The weld cracking tendency of the alloys decreased with decreasing W content and was significantly suppressed with decreasing Si content. In addition, the welding cracking tendency of the Haynes 230 alloy under high energy density was lower than that under low energy density. Although no significant cracking was observed in Weld-E specimens, localized penetration occurred because of the instability of the welding process, which affected the backside roughness of the LPBFed parts. This unacceptable backside roughness was characterized by an excessive root drop-through greater than 0.6 mm and a surface roughness (*R_a_*) exceeding 15 μm. Therefore, subsequent studies mainly focused on the Weld-C and Weld-D specimens [40].

### 3.2. Microstructure of Laser-Welded Joints

**Figure 3:**
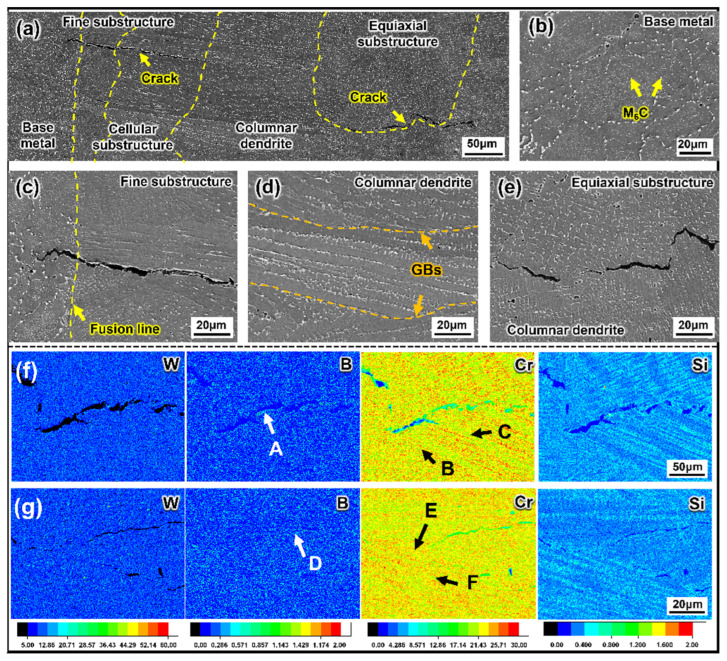
Microstructure morphology of the cracking-sensitive zone of laser-welded joints: (**a**) left side of welded joint; (**b**) base metal zone; (**c**) fusion line and equiaxial substructure zone; (**d**) columnar dendrite zone; (**e**) equiaxial substructure zone; EPMA images of weld cracks in (**f**) 230-A alloy and (**g**) 230-B alloy.

Figure 3 shows the microstructure of the welded joints under the Weld-C process condition, displaying the base metal, weld fine structure, cellular substructure, columnar dendrite, and equiaxial substructure from the outside to the inside. These features were related to the decreasing temperature gradient (*G*) and solidification rate (*V*) during the weld solidification process. Cracking occurred at the planar GBs extending from the base metal to the weld, where elemental segregation was evident, and in the middle of the weld at the boundary between the equiaxial substructure and the columnar dendrites, where liquid metal shrinkage was limited during the final solidification. As shown in Figure 3f,g, further characterization of the EPMA element distribution at the GBs and crack locations in the CZ part of the 230-A and 230-B specimens was performed, and the quantified values are detailed in Table 3, with each condition represented by the statistical mean and standard deviation extracted from *N* = 5 independent measurements. Both sides of the cracks were enriched in element B, which was not evident at the GBs (points B and E) but was higher than that of the matrix. In addition, although W exhibited no apparent macroscopic segregation across the weld body, localized W-rich phases still appeared at specific point-like sites near the cracks where the W content reached up to 18.4 at.% (Point A). Moreover, Si is significantly enriched in cracks and GBs, especially in localized crack locations where blocky Si-rich zones appear [41]. In contrast, Cr had a low content near the cracks and was not the main element affecting cracking. In conclusion, the alloy’s enrichment of W, Si, and B is the key to cracking.

**Figure 4:**
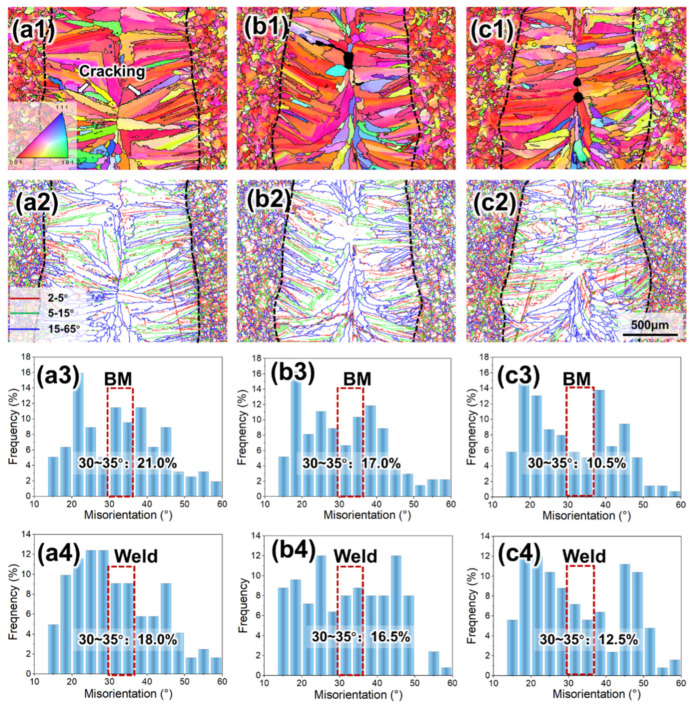
The IPF, grain boundary maps, grain misorientation of BM and weld for three weld joints: (**a1**–**a4**) 230-A, (**b1**–**b4**) 230-B and (**c1**–**c4**) 230-C alloys.

Figure 4 shows the EBSD results of the CZ parts for the three joints under the Weld-C parameter set, where it can be observed that the weld grain orientation is consistent with that of the base metal, with a weak [001] orientation. The average grain sizes of the three base metals are 30.4 ± 10.3 μm, 31.5 ± 12.8 μm, and 30.5 ± 11.4 μm, and the weld grain sizes are 47.2 ± 16.8 μm, 48.4 ± 18.7 μm, and 49.3 ± 21.6 μm, respectively. In Figure 4(a2–c2), a large number of low-angle grain boundaries (LAGB, 2–15°) existed in the base metal, indicating that it was difficult to eliminate the internal dislocations of the LPBFed specimens effectively during the pre-weld heat treatment. The lower percentage of LAGBs in the weld was mainly concentrated near the base metal, where the solidification rate (*V*) was the fastest and the first to solidify, resulting in larger shrinkage stresses. LAGBs were not evident at the cracking location, indicating that stress relief occurred. A functional relationship exists between the grain orientation difference and GB energy [42]. The GB energy is greatest at 30–35° misorientation, where the liquid film at the end of solidification is also the most stable [43]. Compared to alloy 230-A, the percentage of 30–35° misorientation decreased by 4% and 1.5% for the base metal and weld in alloy 230-B, respectively (Figure 4(a3–c3)), while the percentage of 30–35° misorientation decreased by 10.5% and 5.5% for the base metal and weld in alloy 230-C, respectively (Figure 4(a4–c4)). A change in the alloy composition results in grain misorientation and GB energy distribution, which implies varying degrees of GB segregation [44]. Moreover, the grain sizes of the base metal and weld for the three alloys were similar, suggesting that the effect of alloy composition on weld cracking was not due to grain morphology but to GB segregation. Because the grain sizes of the base metal and weld for the three alloys were statistically similar, established literature confirms that when grain morphology variations are negligible, the hot cracking susceptibility is primarily governed by the degree of grain boundary elemental segregation rather than the structural grain morphology [17,45,46].

### 3.3. Precipitation Phases

**Figure 5:**
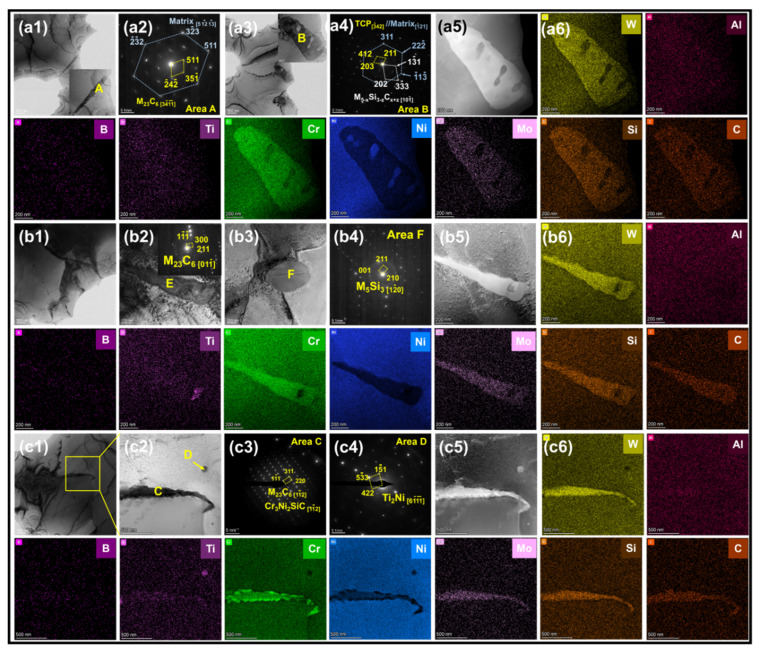
TEM images of microstructure boundaries in three alloys: (**a1**,**a3**) bright-field images, (**a2**,**a4**) selected-area electron diffraction (SAED) results, and (**a5**,**a6**) high-angle annular dark-field (HAADF) images and element distribution of precipitates in 230-A alloy; (**b1**–**b3**) bright-field images, (**b2**,**b4**) SAED results and (**b5**,**b6**) HADDF and element distribution of precipitates in 230-B alloy; (**c1**,**c2**) bright-field images, (**c3**,**c4**) SAED results and (**c5**,**c6**) HADDF and element distribution of precipitates in 230-C alloy.

Figure 5 shows the TEM images of the GB precipitation phases of the CZ part of the three welds. In the 230-A weld, the precipitation phases can be classified into two types: the rod-shaped M_23_C_6_ carbide and a massive mixed phase consisting of a topologically close-packed (TCP) phase exhibiting a rhombohedral μ-phase crystal structure TCP phase alongside silicides. As shown in Figure 5(b1–b6), two precipitation phases were also present in alloy 230-B, namely, strip M_23_C_6_ carbides and massive M_5_Si_3_ silicides, and there was also a localized Ti-rich region within the strip phase. Figure 5(c1–c6) shows that the precipitation phase of alloy 230-C mainly consisted of M_23_C_6_ and complex Cr_3_Ni_2_SiC carbosilicides, a phase known to form stably in silicon-modified nickel environments [47]. The interfacial mismatch between the precipitated phase and substrate was calculated using (1) [48], as shown in Table 4, referencing the TEM data from Figure 5.

(1) $$\eta =2\left|\frac{nd_{matrix}-md_{precipitates}}{md_{matrix}+nd_{precipitates}}\right|$$

where $d_{matrix}$ and $d_{precipitates}$ are the crystal spacings of the matrix and M_12_C, respectively. *m* and *n* can be identified by $d_{matrix}/d_{precipitates}=m/n$ (*m* and *n* are minimum integers).

The interface between M_23_C_6_ and the matrix is a good semi-coherent interface with an interfacial mismatch of less than 0.3% or even a coherent interface [3]. The interface between the carbides and matrix is serrated, which provides sound mechanical occlusion. The mismatches of the TCP phase and silicide with the matrix were calculated to be <0.7% and <0.9%, respectively, and the interface with the matrix was straight and smooth with a large interfacial strain (Figure 5(a1–a6)). The main nonmetallic elements in the three weld segregations were C and Si. In contrast, no significant enrichment of B occurred, which indicates that in the uncracked boundary, a large amount of substitution of C did not reduce the melting point of the carbides [49].

**Figure 6:**
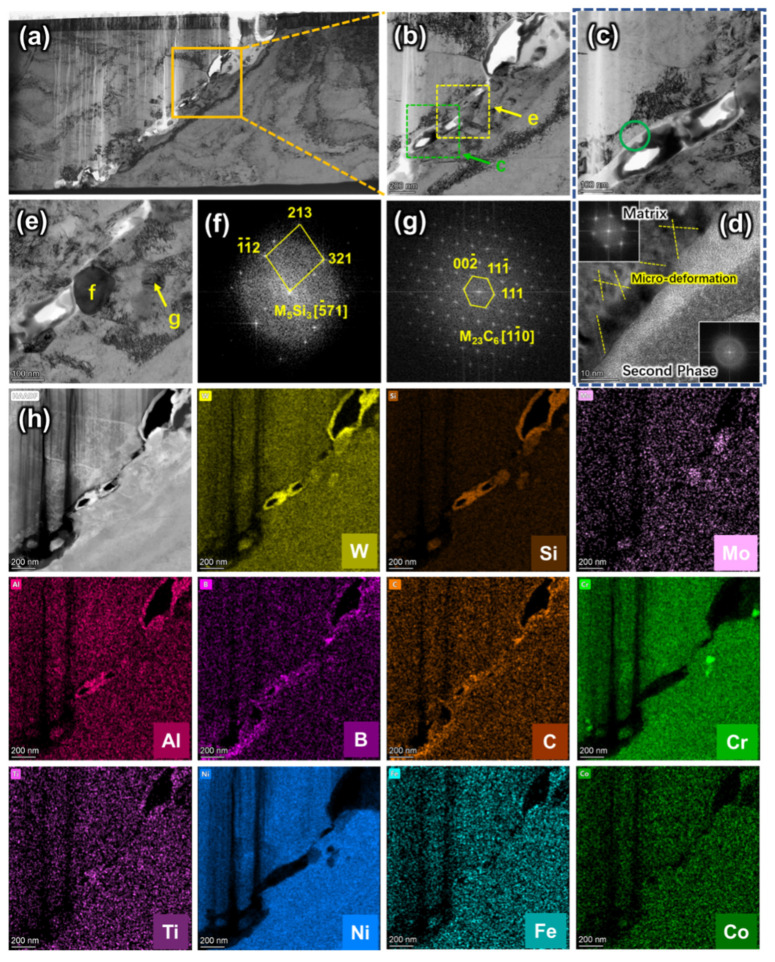
TEM results of welding crack: (**a**–**c**,**e**) bright-field images; (**d**) high-resolution transmission electron microscope (HRTEM) images of green circle in (**c**); (**f**,**g**) SAED images of areas f and g in (**e**); (**h**) HAADF and elemental distribution images.

Figure 6 shows the TEM microstructure of the 230-B weld cracks. In contrast to the elemental segregation at the GBs shown in Figure 5, the elements on both sides of the crack exhibit a regional distribution. The higher W, Si, and Al contents in the precipitation phases on both sides of the crack, which were continuously cracked and not yet fully cracked, were the key to cracking. From the elemental distribution trend, the formation of W-, Si-, Al-rich phases finally formed at the GBs, indicating that the phase had a low melting point and was prone to forming a stable liquid film at the end of solidification, which broke up within them. The sides of the crack also contained small amounts of Mo- and W-rich silicides (M_5_Si_3_) and Cr-rich carbides (M_23_C_6_) away from the crack. In terms of the distance from the compounds to the cracking, the Cr-rich carbides precipitated first with a higher melting point than the silicides [45]. Moreover, B overlapped with C and formed low-melting-point compounds with the other elements.

The quantitative interfacial misfit (δ) compiled in Table 4 provides a fundamental correlation between the crystallographic characteristics of the precipitates and the hot cracking susceptibility of the grain boundaries (GBs). Thermodynamically, the interfacial energy (*γ_int_*) of a secondary phase precipitate is highly sensitive to its lattice coherence with the matrix. The M_23_C_6_ carbide exhibits a strict cube-on-cube orientational relationship with the *γ*-matrix, yielding an exceptionally low lattice misfit ranging from 0.17% to 0.29%. This ultra-low misfit signifies a highly coherent/semi-coherent interface, which minimizes both the local elastic strain energy and the interfacial tension, thereby forming a strong, crack-resistant metallurgical bond. In stark contrast, the topologically close-packed (TCP) μ-phase and the massive silicides exhibit significantly higher structural misfits with the matrix (0.71–0.89% and 0.64%, respectively). These large crystallographic deviations force the formation of incoherent or highly disordered semi-coherent phase boundaries. According to Eshelby’s inclusion theory, such severe lattice distortion generates massive localized elastic strain fields and stress concentrations at the interfaces. Under the influence of severe tensile thermal shrinkage stress during the terminal stages of welding solidification, these highly strained incoherent boundaries act as ideal stress-raisers. Consequently, the high interfacial energy and severe local strain concentration destabilize the intergranular liquid films, facilitating interfacial de-cohesion and separation. This clarifies why the propagation of intergranular cracks is strictly linked to the presence of incoherent TCP μ-phases and silicides, whereas coherent M_23_C_6_ carbides effectively suppress liquid film rupture.

## 4. Discussions

Solidification cracking is mainly affected by the localized shrinkage stresses on both sides of the liquid film and the elemental segregation of the alloy at the end of solidification. The solidification sequence of the weld determines shrinkage stress, whereas elemental segregation is related to the solidification rate and initial alloy composition. In this section, the flow behavior of the molten pool and the elemental migration process during solidification are discussed based on the above results, so as to reveal the inhibition mechanism of weld cracking by elemental modulation.

### 4.1. Preferential Solidification Driving Cracking in the CZ

**Figure 7:**
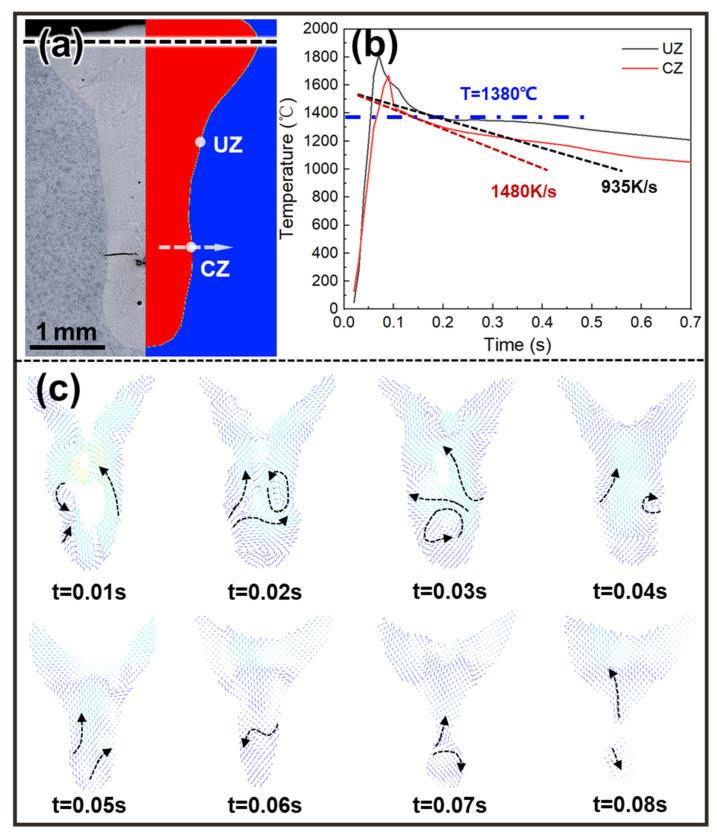
Simulation of the temperature field and melt pool flow of laser-welded Haynes 230 alloy: (**a**) cross-sectional morphology and simulation results of the weld; (**b**) temperature profiles near the fusion line in the cracking-sensitive zone (CZ) and the uncracked zone (UZ) of the weld; (**c**) direction of flow of the melting pool cross-section.

The tendency to crack was more significant at lower heat inputs when the depth of fusion was smaller, as shown in Figure 2. At a higher heat input, the depth of fusion increased, and weld penetration occurred, at which time cracking was eliminated. However, regardless of the weld type, the relative positions of the cracks in the weld and the cracking direction varied less, and cracking appeared in the CZ part of the deep melt zone. The melt pool flow behavior during the welding process was simulated to clarify the difference in solidification behavior between the UZ and CZ during the welding process. Figure 7a compares the weld cross-section and the melt pool cross-section with good similarity. Similar weld morphologies and simulation results were found by Mondal et al. [11], indicating that the simulation results were highly accurate in terms of melt pool generation. To account for model accuracy and validation uncertainty, a sensitivity analysis was performed by varying the boundary heat transfer conditions by ±5%. This variation altered the predicted absolute temperature distribution by approximately ±35 K and the local cooling rates by ±45 K/s, confirming that the model remains highly stable and representative under standard assumptions.

The molten-pool flow behavior after laser scanning was further analyzed, as shown in Figure 7c. During the laser welding process, the keyhole was unstable with obvious fluctuations, and the liquid metal flowed around the keyhole. This was mainly due to the molten pool’s forced flow, which was caused by the keyhole’s fluctuations with a large flow rate. With the gradual disappearance of the keyhole, the transversal flow of the melt pool with a larger flow velocity gradually appeared in the CZ parts of the weld, while the flow vortex with a small flow velocity appeared at the bottom of the melt pool (Δt = 0.01~0.02 s). The flow in the weld pool gradually changed from the strong convective behavior caused by the laser-induced steam recoil force to the surface tension-induced Marangoni flow (Δt = 0.03~0.04 s). The welded bottom and lower-middle melt pool edges maintained a low melt-pool flow rate, whereas the weld center still had a high flow rate owing to the higher temperature, which led to further solidification at the weld edges. In the middle of solidification, the transverse flow of liquid metal in the CZ part of the weld reappeared, mainly influenced by surface tension (Δt = 0.05). The liquid metal flowed gradually from the side wall of the CZ part to the UZ part and the bottom of the weld, indicating that the temperature of the side wall of the CZ part was lower, and the flow of the molten pool took away the heat from the CZ part, causing it to solidify preferentially (Δt = 0.06~0.07).

The temperature curves of the melt pool boundaries in the CZ and UZ were extracted, as shown in Figure 7b. The temperature gradient in the CZ part was calculated to be approximately 1.16 × 10^6^ K/m, and the average cooling rate during solidification was approximately 1480 K/s. The temperature gradient in the UZ was approximately 9.2 × 10^5^ K/m, and the average cooling rate during solidification was approximately 935 K/s. The larger *V* caused the CZ to solidify preferentially, which could result in the liquid film at the GBs at the end of solidification being subjected to solidification shrinkage stresses in the UZ and bottom weld [50]. Consequently, the GB liquid film in the CZ breaks down, increasing the cracking driving force of the CZ part.

To evaluate the fidelity and boundary limitations of the three-dimensional heat transfer and fluid flow model, both geometric validation and an analysis of computational assumptions were addressed. Due to the extreme thermal gradients (peak temperatures > 2500 K) and the optical opacity of the liquid metal pool during laser deep fusion welding, direct in situ measurement of internal flow velocities is experimentally restricted. Consequently, in accordance with established computational welding mechanics, the macroscopic fusion boundary profile was adopted as the primary validation benchmark. The simulated molten pool morphology displays excellent quantitative congruence with the experimental cross-section in Figure 7a, validating the accuracy of the configured driving forces (recoil pressure, surface tension, and buoyancy).

The mathematical formulations operate under two primary simplified physical assumptions: (i) the omission of laser-induced plasma attenuation, and (ii) a laminar Newtonian fluid regime. The omission of plasma shielding is physically justified because the high-brightness fiber laser wavelength (λ = 1.06 μm) exhibits negligible inverse Bremsstrahlung absorption within the vapor plume, unlike CO_2_ laser systems. Concurrently, the laminar flow assumption is standard for micro-scale molten zones (<2 mm), ensuring robust extraction of macro-scale thermal vectors. Although minor calculation uncertainties (±5–8%) inevitably arise from grid discretization and temperature-dependent thermophysical property scaling via JmatPro, they do not deviate from the relative fluid kinetic trends. Because the underlying crack-initiation mechanism discussed herein is governed by the relative cooling rate differential between the CZ and UZ rather than absolute localized temperatures, the inherent model uncertainty does not compromise the validity of the conclusions.

### 4.2. Solidification Cracking Mechanism at Weld GBs

Massive segregation of alloying elements is an essential cause of the formation of low-melting-point compounds at the GBs, thereby increasing the tendency to crack during solidification. The above results reveal that segregating W, Si, and Al is essential for GB cracking. Previous studies indicated that weld solidification cracking in similar superalloys is closely associated with the precipitation of W-, Si-, and Al-rich TCP phases and M_5_Si_3_ silicides [28]. Liu et al. [17,51,52] suggested that Al, Ti, W, and Mo-rich Laves-phase and γ’-phase eutectics led to the hot cracking. These conclusions are similar to the elemental segregation results obtained in the present study. In addition, near the interface between the second phase and the matrix, a large number of stacking faults appeared within the matrix (Figure 6d), indicating that microdeformation occurred during the solidification process. Moreover, as listed in Table 4, a larger mismatch between silicides and matrix promoted the strain concentration at GBs, promoting the cracking of the W-, Si-, and Al-rich low-melting-point liquid film. Although the precipitated phase in Figure 6d exhibits an amorphous structure, it is speculated that it was obtained from the transition of the TCP phase during ion thinning based on the phenomenon of weld cracking reduction with a decrease in the TCP phase and its elemental composition. In addition, B and C usually possess greater diffusion rates owing to their smaller atomic diameters. They are prone to forming low-melting-point compounds by massive segregation at the GBs, which increases the liquid film stability and solidification cracking tendency at the end of solidification [15,49].

### 4.3. Elemental Migration Behavior During Precipitation Phase Formation

Based on the above discussion, elemental distribution plays a key role in grain boundary cracking. Uneven diffusion and solute distribution during solidification lead to segregation. During the solidification process of laser welding, the solidification rate reached 10^3^ K/s, accelerating the nucleation of dendrites and keeping the segregation within a small range [53]. The solid–liquid interface movement rate was consistent with the welding rate, and the effect of the dynamics on the microstructure growth and segregation became significant. Solute retention occurred during solidification, which caused the solidification interface solute distribution to deviate from the equilibrium state, leading to significant segregation between the dendrites [54]. Because the solidification rate influences the solute partition coefficient for rapid solidification, a further influence on subsequent solute partitioning is expected, especially on the location of the final solidified GBs. To understand the behavior of elemental segregation during solidification, the elemental distribution in the solid and liquid phases during solidification was comprehensively discussed from the TD perspective, enabling a better understanding of the precipitated phase formation.

Because of the intensive convection effect of the laser welding process, the liquid molten pool can be regarded as homogeneous. In addition, solute retention caused a large solute enrichment in the solid phase along the solidification front, which allowed the reverse diffusion of some of the solute elements in the solid phase into the liquid phase. Based on the two solute behaviors, the traditional Scheil model [55] is no longer appropriate, and the Clyne and Kurz model can more closely describe the solute distribution behavior, which better matches the rapid solidification process of laser welding [56]:

(2) $$C_{S}=kC_{0}{\left[1-(1-2\alpha^{\prime }k)f_{s}\right]}^{\frac{k-1}{1-2\alpha^{\prime }k}}$$

where *C*_S_ is the solute concentration in the solid phase, *k* is the equilibrium solute partition coefficient, *C*_0_ is the initial alloy composition, and *f*_s_ is the solid fraction.

(3) $$\alpha^{\prime }=\frac{D_{s}t_{f}}{\lambda_{f}^{2}}(1-e^{-\frac{1}{\alpha }}-0.5e^{-\frac{1}{2\alpha }})$$

(4) $$t_{f}=\frac{\Delta T}{GV}$$

where *λ*_f_ is the final dendrite arm spacing, Δ*T* is the undercooling at the solidification interface, *G* is the temperature gradient, and *V* is the solidification rate. *D*_s_ is the solute diffusion coefficient in the solid phase, which can be calculated as Formula (5) [56]:

(5) $$D_{S}=D_{0}e^{-\frac{Q}{RT}}$$

where *D*_0_ and *Q* (activation energy) are listed in Table 5, *R* is the gas constant (8.314 J/(mol-K)), and *T* is the temperature (K).

Burden and Laxmanan modeled dendritic tip supercooling as consisting of thermal supercooling, dendrite growth concentration gradient supercooling, the Gibbs–Thomson effect due to the radius of curvature of the dendrite tip, and dynamical supercooling, as shown in Formula (6) [46,61]:

(6) $$\Delta T=\frac{D_{L}G}{V}-\frac{VmC_{0}(1-k)r}{D_{L}}-krG+\frac{2\sigma T_{L}}{\rho \Delta H_{f}r}$$

where *D*_L_ is the solute diffusion coefficient in the liquid phase, m is the equivalent equilibrium liquidus slope, *r* is the radius of a dendrite tip, *σ* is the surface tension at the solid–liquid interface, *T*_L_ is the liquidus temperature, *ρ* is the density of the solid phase, and Δ*H*_f_ is the latent heat (about 180 J/g, obtained by JMatPro). Owing to the solute trapping phenomenon that occurs during solidification when the solute partition coefficient is affected by the solidification rate (*V*), the equilibrium partition coefficient (*k*_i_′), the slope of the liquid phase line (*m*′), and liquid phase composition along the solidification front (*C*_L_) are modified as follows:

(7) $${k_{i}}^{\prime }=\frac{k_{i}+a_{i}VD_{Li}^{-1}}{1+a_{i}VD_{Li}^{-1}}$$

(8) $$m^{\prime }=m\frac{1-k^{\prime }[1-\ln(k^{\prime }/k)]}{1-k^{\prime }}$$

(9) $$C_{L}=\frac{C_{0}}{1-(1-k^{\prime })Iv(P)}$$

(10) $$Iv(P)=Pe^{P}\int_{P}^{\infty }\frac{e^{-x}}{x}dx$$

where *k*_i_ is the equilibrium partition coefficient of solute *i* in a complex multicomponent system, *a*_i_ is the atomic dimension of element *i*, and *D*_Li_ is the diffusion coefficient of solute *i* in the liquid phase, which can be calculated using Ref. [62]. *P* is the Peclet number of the solute related to the dendrite growth rate and dendrite spacing [63], and Iv(*P*) is the Ivantsov function of the solute Peclet number.

**Figure 8:**
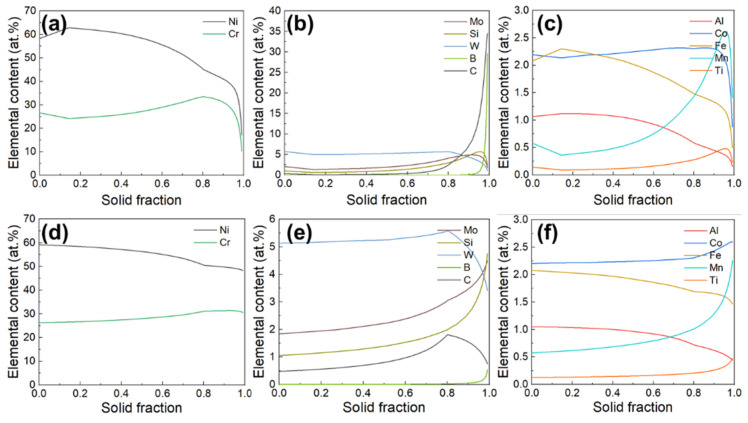
Elemental distribution along the solidification front in the crack-sensitive area: (**a**–**c**) solid phase and (**d**–**f**) liquid phase.

**Figure 9:**
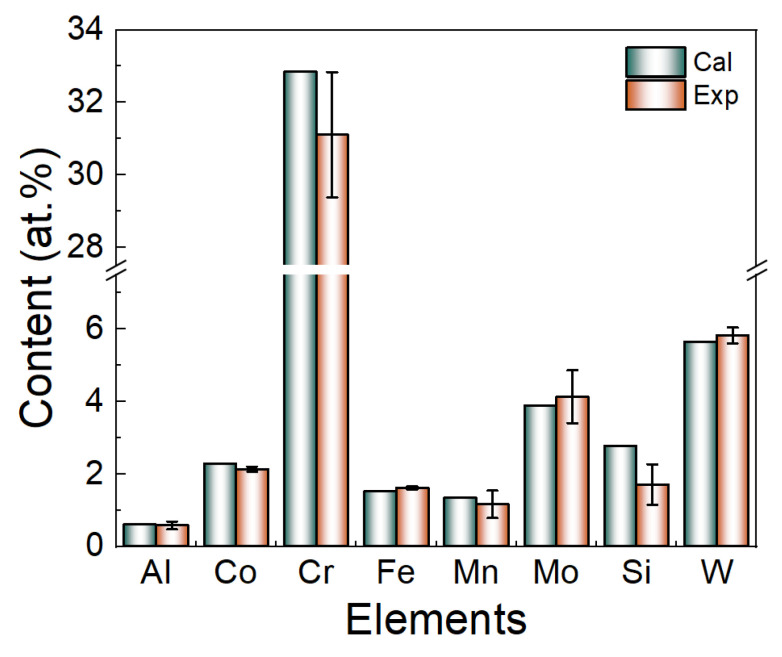
Calculated elemental distributions at the end of solidification (Cal) and average EDS point scans of measured grain boundaries (Exp) in the CZ parts.

The supercooling of the CZ part was calculated as 4.892 K based on *V* and *G* in Section 4.1, where the dynamic supercooling of 4.142 K was the main contributor. By further combining Formulas (4)–(10), the behavior of the solute distribution at the solid- and liquid-phase solidification fronts at the CZ part was calculated, as shown in Figure 8. The calculated elemental distribution at the end of solidification was similar to the results of the GB elemental segregation presented in Figure 9, suggesting that the calculation process was in good agreement with the actual solidification process.

Based on the calculated solidification path, the mechanism of solute element migration along the solidification front can be elucidated (schematically summarized in Figure S1). In the early stages of solidification, a high-melting-point Ni-based solid solution preferentially nucleates, while laser-induced strong convection maintains a relatively homogeneous liquid phase. As solidification proceeds, cellular and dendritic substructure growth progressively restricts inter-dendritic fluid flow, leading to the decay of laser-driven convection. Consequently, solute enrichment and severe solute trapping aggravate at the solidification front. In the terminal stages (0.8 < *f_s_* < 1.0), the primary precipitation of M_23_C_6_ induces a successive depletion of C and Cr in the remaining liquid, followed by a localized drop in W content owing to TCP and silicide formation (Figure 5 and Figure 6). Because of the low solid-state diffusion coefficient of W, negligible reverse diffusion occurs after the terminal precipitation of these trace phases. Concurrently, interstitial atoms like B and C, which possess significantly higher diffusion rates, easily partition into the final liquid film. This terminal co-segregation severely expands the low-melting-point mushy zone at the grain boundaries, promoting the formation of highly detrimental, low-melting-point M_x_(B, C)_y_ phases rather than stable carbides, which ultimately triggers solidification cracking.

### 4.4. Element Migration Alteration by Compositional Modulation at the Solidification Front

Alterations in the alloy composition led to variations in the composition of the GB precipitation phases. In Figure 5 and Figure 6, the GB precipitates can be mainly categorized into Cr-rich M_23_C_6_; W-, Al-, and Si-rich TCP phases; Cr-, W-, Mo-, and Si-rich silicides; and C-and B-enriched zones that are susceptible to the formation of low-melting-point compounds. Table 6 provides the formation-free energies of the compounds under the temperature conditions at the end of solidification, which reveal a greater tendency for the formation of the W-Al-Si phase and M_5−x_Si_3−z_C_x+z_, in agreement with the results of the present study. The tendency of non-equilibrium solidification cracking during laser welding depends mainly on the number of different types of compounds precipitated, which in turn is influenced by the elemental content of the liquid phase. As discussed in Section 4.3, the diffusion coefficient (*D*) and partition coefficient (*k*) of the solute elements in the Ni matrix directly affect the degree of segregation. The solidification path is significantly affected by variations in the initial composition of the alloy, which lead to fluctuations in *D* and *k* influenced by the temperature and compositional trends. However, the effect of the alloy composition change on solute elemental segregation is difficult to observe directly, whereas TD theoretical calculations can provide the diffusive behavior of the elemental distributions during solidification.

**Figure 10:**
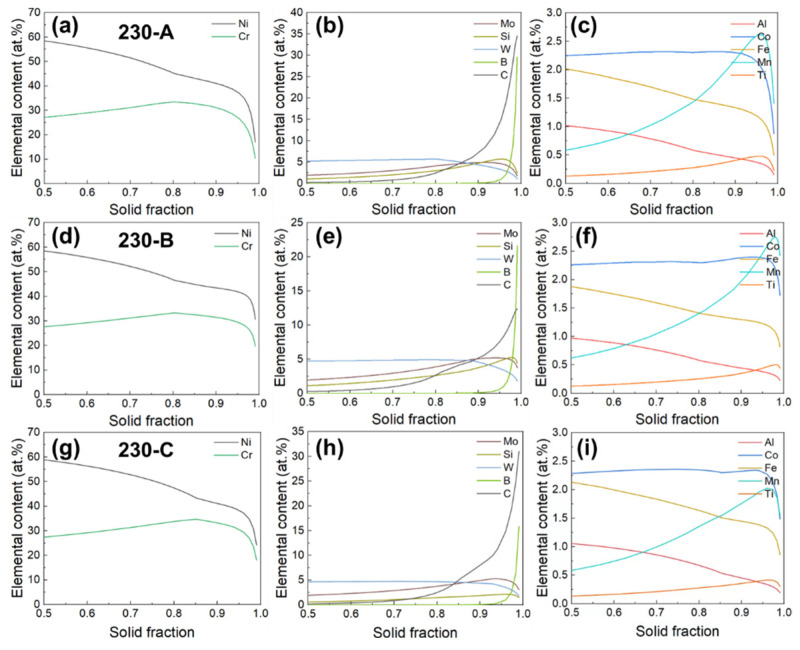
Variation in elemental content in the solid phase (0.5 < *f*_s_ < 1.0) along the solidification front of (**a**–**c**) 230-A, (**d**–**f**) 230-B, and (**g**–**i**) 230-C alloys.

**Figure 11:**
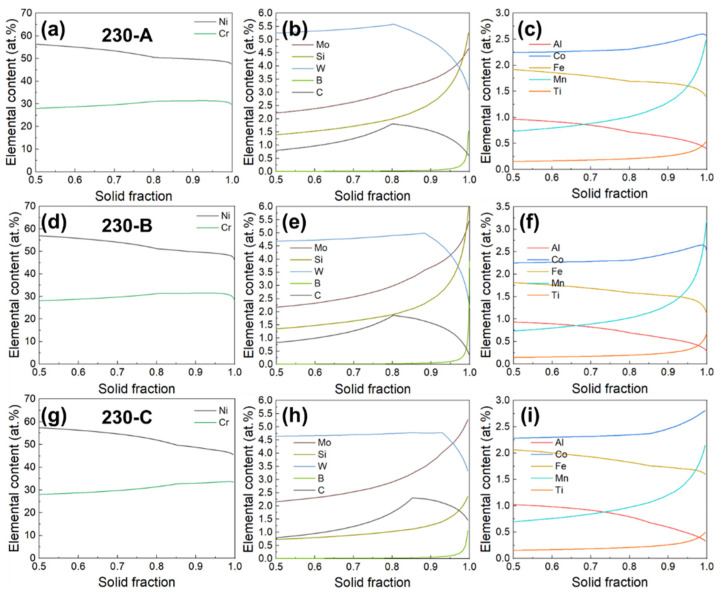
Variation in elemental content in the liquid phase (0.5 < *f*_s_ < 1.0) along the solidification front of (**a**–**c**) 230-A, (**d**–**f**) 230-B, and (**g**–**i**) 230-C alloys.

Figure 10 and Figure 11 present the behavior of solute element enrichment during the late and end solidification processes, including the theoretical element distributions for the three alloys in the solid and liquid phases with *f*_s_ ranging from ~0.5 to ~1.0. The trends of the different elemental distribution curves for the three alloys are more or less the same, whereas the main differences appear at the late solidification stage (0.8 < *f*_s_ < 0.9) and the end of solidification (0.9 < *f*_s_ < 1.0). In the late and final stages of solidification, compared to alloy 230-A, the Cr content of alloy 230-B increased in the solid phase and varied less in the liquid phase, implying that alloy 230-B preferentially precipitated in a Cr-rich phase. The delayed reduction in W content, delayed inflection point of W content in the liquid phase, and lower content of Si in the solid and liquid phases at the end of solidification indicated that alloy 230-B exhibited a lower tendency to produce W-Si agglomerated compounds. Consequently, the cracking tendency was attenuated, as shown by the comparison of the elemental distribution at the cracking location for the high-W alloy (14.33 wt.%) in the previous study [28] and in Figure 6. The C content in the solid phase of alloy 230-B showed a small change during the late solidification period and a significant decrease at the end. In the liquid phase, the C content varied less, suggesting that higher-melting-point carbides were precipitated; however, the amount of carbon-rich, low-melting-point compounds that precipitated during the final solidification decreased. The high overlap of the C and B elemental enrichment positions in Figure 6 also illustrates that alleviating C enrichment could prevent severe B enrichment, thereby preventing the formation of C and B low-melting-point compounds or eutectics. Comparing the 230-B and 230-C alloys in the late and final stages of solidification, the solid phase of the 230-C alloy contained less Cr and Ni, more C, and less B, Mo, and W with delayed inflection points for the W shift. In the liquid phase, the Cr, Ni, and Mo contents were lower, while the C content was higher. The inflection points for C and Cr were synchronously delayed, the W content was higher, and the inflection points were further delayed. Therefore, at the end of solidification, high-melting-point carbides of Cr and W were preferentially precipitated, with Cr_23_C_6_ precipitating first, followed by W-rich carbides. In the solid phase, no obvious Si segregation occurred, and the inflection point of the transition was further delayed. In the liquid phase, the Si content remained low without obvious segregation, which inhibited the precipitation of silicides and low-melting-point W-Si-Al phases.

In summary, the reduction in the W content in the alloy promoted the simultaneous reduction in W, Si, and C precipitation in the solid phase at the end of solidification. The generation of W-, Si-, and Al-rich TCP phases was suppressed, and the enrichment of C and B in the final solidification was avoided, thereby reducing the tendency for weld cracking. Furthermore, the further reduction in the Si content in the alloy resulted in a reduction in W and Si enrichment and a delay in the transition inflection point, thus delaying the precipitation of the second phase. Carbon in the solid and liquid phases was maintained at a high content to promote high-melting-point carbide precipitation while inhibiting the production of silicides, further improving the ability of the alloy to resist cracking.

## 5. Conclusions

The laser weld cracking behavior of LPBFed Haynes 230 alloy was investigated. Weld cracking was suppressed by modulating the alloy composition. Theoretical calculations of the elemental segregation behavior at the end of solidification and the mechanism of precipitated phase formation were revealed using thermal dynamics, and the specific conclusions were as follows:

(1) The weld cracking susceptibility of the LPBFed alloy is strongly correlated with the presence of low-melting-point W-, Si-, and Al-rich TCP phases (possessing a rhombohedral μ-phase crystal structure) and silicides. These secondary phases introduce high interfacial mismatches (0.64–0.89%) and localized elastic strain concentrations along the grain boundaries. Modulating the initial W and Si contents successfully transitions these detrimental precipitates into stable, high-melting-point carbides, effectively suppressing crack initiation.
(2) Three-dimensional fluid dynamics and thermal simulations reveal that the cracking-sensitive zone (CZ) solidifies preferentially under an accelerated local cooling rate (~1480 K/s) driven by directional molten pool flow. This preferential solidification leaves the remaining intergranular liquid films highly vulnerable, subjecting them to severe tensile thermal shrinkage stresses from the adjacent lagging solidification zones and providing a strong mechanical driving force for crack propagation.
(3) Reducing the initial W content minimizes the degree of W-Si co-segregation at the solidification front. Further reduction in the Si content to 0.25 wt.% successfully delays the elemental transition inflection points during terminal solidification (0.8 < *f_s_* < 1.0), comprehensively suppressing low-melting-point silicide formation and allowing high-melting-point carbides to dominate, which successfully optimizes the weldability and eliminates macroeconomic cracks in the additively manufactured superalloy.

## Figures and Tables

**Table 1:** Chemical composition of three Haynes 230 alloy powders (wt.%).

Element	C	Si	Mn	W	Cr	Mo	Fe	Co	Al	B	Ti	Ni
Casting	0.092	0.49	0.50	14.98	21.66	2.79	1.88	2.03	0.46	<0.001	0.093	Bal.
230-A	0.091	0.47	0.50	14.96	21.64	2.80	1.84	2.06	0.45	<0.001	0.095	Bal.
230-B	0.096	0.47	0.51	13.62	22.02	2.77	1.76	2.01	0.44	<0.001	0.090	Bal.
230-C	0.092	0.25	0.48	13.56	21.71	2.76	1.89	2.10	0.47	<0.001	0.095	Bal.

**Table 2:** Five process parameters of laser welding.

Process Parameters	Weld-A	Weld-B	Weld-C	Weld-D	Weld-E
Laser power (W)	2000	2700	3100	3100	3500
Scanning speed (m/s)	0.04	0.04	0.04	0.02	0.02
Line energy density (J/mm)	50.0	67.5	77.5	155.0	175.0

**Table 3:** EMPA point-scan results of different joints for 230-A and 230-B alloys (at.%, based on *N* = 5 independent measurements).

Elements	W	B	Cr	Si
Point A (Cracking GB)	18.4 ± 0.6	0.63 ± 0.05	21.7 ± 0.8	0.70 ± 0.06
Point B (Uncracking GB)	14.1 ± 0.1	0.52 ± 0.04	28.0 ± 0.9	0.37 ± 0.04
Point C (Subgrain boundary)	11.6 ± 0.4	0.38 ± 0.03	24.5 ± 0.7	0.44 ± 0.01
Matrix (230-A)	6.48 ± 0.12	0.14 ± 0.01	20.3 ± 0.5	0.19 ± 0.02
Point D (Cracking GB)	10.6 ± 0.4	0.4 ± 0.04	19.3 ± 0.6	0.49 ± 0.04
Point E (Uncracking GB)	9.9 ± 0.2	0.37 ± 0.01	24.7 ± 0.8	0.37 ± 0.03
Point F (Subgrain boundary)	9.8 ± 0.5	0.32 ± 0.02	26.2 ± 0.7	0.36 ± 0.03
Matrix (230-B)	6.1 ± 0.18	0.1 ± 0.01	19.5 ± 0.4	0.22 ± 0.02

**Table 4:** Interfacial misfit between the precipitated phase and the matrix in Figure 5.

Precipitated Phase	Orientational Relationship	d_m_ (nm)	d_p_ (nm)	m	n	Misfit (%)
M_23_C_6_	(511)_Matrix_//(511)_M23C6_ [5$\bar{12}\bar{13}$]_Matrix_//[3$\bar{4}\bar{11}$]_M23C6_	0.0692	0.2052	1	3	0.29
	(23$\bar{2}$)_Matrix_//(35$\bar{1}$)_M23C6_ [5$\bar{12}\bar{13}$]_Matrix_//[3$\bar{4}\bar{11}$]_M23C6_	0.0895	0.1802	1	2	0.17
M_5−x_Si_3−z_C_x+z_	($\bar{3}\bar{1}\bar{1}$)_Matrix_//(202)_M5−xSi3−zCx+z_ [$\bar{1}$21]_Matrix_//[10$\bar{1}$]_M5−xSi3−zCx+z_	0.1088	0.1860	3	5	0.64
TCP	(22$\bar{2}$)_Matrix_//(01$\bar{2}$)_TCP_ [$\bar{1}$21]_Matrix_//[$\bar{3}42$]_TCP_	0.1042	0.2158	1	2	0.89
	(1$\bar{1}$3)_Matrix_//(203)_TCP_ [$\bar{1}$21]_Matrix_//[$\bar{3}42$]_TCP_	0.1088	0.2820	3	8	0.71

**Table 5:** Diffusion coefficients of elements in solid nickel [57,58,59,60].

Element	B	C	Al	Ti	Cr	Mo	W	Si
D_0_ (m^2^/s)	3.27 × 10^−4^	3.70 × 10^−5^	8.79 × 10^−5^	1.07 × 10^−4^	9.25 × 10^−5^	1.09 × 10^−5^	6.90 × 10^−6^	2.90 × 10^−5^
Q (kJ/mol)	163.56	147.08	276.0	276.91	299.0	278.8	297.1	259.54

**Table 6:** Gibbs free energy (G_f_) at 1320 °C of precipitates in grain boundary (kJ/mol).

Precipitate	Cr_23_C_6_	Mo_23_C_6_	W_23_C_6_	Cr_5_Si_3_	Mo_5_Si_3_	W_5_Si_3_
G_f_	−35.15	−33.96	−31.4	−34	−35.18	−31.3
Precipitate	Cr_23_B_6_	W_23_B_6_	Mo_23_B_6_	Cr_3_B_2_	W_3_B_2_	Mo_3_B_2_
G_f_	−26.72	−24.03	−25.55	−32.7	−25.31	−27.03
Precipitate	Cr_23_B_6_	W_23_B_6_	Mo_23_B_6_	Cr_3_B_2_	W_3_B_2_	Mo_3_B_2_
G_f_	−26.72	−24.03	−25.55	−32.7	−25.31	−27.03
Precipitate	Cr_5−x_Si_3−z_C_x+z_	W_5−x_Si_3−z_C_x+z_	Mo_5−x_Si_3−z_C_x+z_	Ni_2_Ti	Ni_3_Ti	W-Si-Al
G_f_	−38.78	−33.89	−37.35	−26.77	−25.85	−34.83
Precipitate	P-Ni_40_Cr_18_Mo_42_	μ-Fe_7_W_6_	σ-FeMo	R-Cr_18_Mo_31_Co_51_	W(Si, Al)_2_	
G_f_	−18.8	−8.81	−10.24	−16.6	−32.23	

## Data Availability

The original contributions presented in this study are included in the article/Supplementary Material. Further inquiries can be directed to the corresponding author.

## Supplementary Materials

The following supporting information can be downloaded at https://www.mdpi.com/article/10.3390/ma19153210/s1, Figure S1: Schematic diagram of solute element migration during solidification.

## References

[1] Wu Z., Yarasi S.R., Seo J., Lamprinakos N., Rollett A.D. (2022). Study of the Printability, Microstructures, and Mechanical Performances of Laser Powder Bed Fusion Built Haynes 230. Metals.

[2] Hrutkay K., Kaoumi D. (2014). Tensile deformation behavior of a nickel based superalloy at different temperatures. Mater. Sci. Eng. A.

[3] Liu M., Lin D., Zhang Y., Wang D., Xiao B., Xu L., Han Y., Minami F. (2025). Excellent mechanical properties and superelasticity: Bimodal heterostructure enhances NiTi alloy fabricated via laser powder bed fusion. Int. J. Plast..

[4] Lam M.C., Lim S.C.V., Song H., Zhu Y., Wu X., Huang A. (2022). Scanning strategy induced cracking and anisotropic weakening in grain texture of additively manufactured superalloys. Addit. Manuf..

[5] Hu X., Xue Z., Zhao G., Yun J., Shi D., Yang X. (2019). Laser welding of a selective laser melted Ni-base superalloy: Microstructure and high temperature mechanical property. Mater. Sci. Eng. A.

[6] Rautio T., Mäkikangas J., Kumpula J., Järvenpää A., Hamada A.S. (2021). Laser Welding of Laser Powder Bed Fusion Manufactured Inconel 718: Microstructure and Mechanical Properties. Key Eng. Mater..

[7] Caiazzo F., Alfieri V. (2021). Optimization of laser beam welding of steel parts made by additive manufacturing. Int. J. Adv. Manuf. Technol..

[8] Han C., Jiang P., Geng S., Ren L. (2024). Multi-physics multi-scale simulation of unique equiaxed-to-columnar-to-equiaxed transition during the whole solidification process of Al-Li alloy laser welding. J. Mater. Sci. Technol..

[9] Xin J., Wang W., Yang X., Boubeche M., Wang S., Zhang H., Huang C., Li Y., Lyu B., Shen F. (2023). Dissimilar laser welding of CrMnFeCoNi high entropy alloy and 316LN stainless steel for cryogenic application. J. Mater. Sci. Technol..

[10] Taheri M., Razavi M., Kashani-Bozorg S.F., Torkamany M.J. (2021). Relationship between solidification and liquation cracks in the joining of GTD-111 nickel-based superalloy by Nd: YAG pulsed-laser welding. J. Mater. Res. Technol..

[11] Mondal B., Gao M., Palmer T.A., DebRoy T. (2022). Solidification cracking of a nickel alloy during high-power keyhole mode laser welding. J. Mater. Process. Technol..

[12] Tian Z., Chen S., Wang Y., Tao W., Ye X., Li N., Ren W., Yang S. (2023). Laser welding of GH3539 alloy for molten salt reactor: Processing optimization, microstructure and mechanical properties. Mater. Charact..

[13] Chen S., Ye X.-X., Tsang D.K.L., Jiang L., Yu K., Li C., Li Z. (2019). Welding solidification cracking susceptibility and behavior of a Ni-28W-6Cr alloy. J. Mater. Sci. Technol..

[14] DuPont J.N., Robino C.V., Anderson T.D. (2008). Influence of Gd and B on solidification behaviour and weldability of Ni–Cr–Mo alloy. Sci. Technol. Weld. Join..

[15] Ernst S.C. (1994). Weldability Studies of Haynes^®^ 230 Alloy, Weld. J. Weld. Res. Suppl..

[16] Liu X., Hu R., Zou H., Yang C., Luo X., Bai J., Ma R. (2023). Investigation of cracking mechanism and yield strength associated with scanning strategy for an additively manufactured nickel-based superalloy. J. Alloys Compd..

[17] Liu X., Hu R., Luo X., Yang C., Gao X. (2022). A high-strength Ni–Cr–W based superalloy prepared by laser powder bed fusion: Printability, microstructure and tensile properties. Mater. Sci. Eng. A.

[18] Gruber H., Hryha E., Lindgren K., Cao Y., Rashidi M., Nyborg L. (2022). The effect of boron and zirconium on the microcracking susceptibility of IN-738LC derivatives in laser powder bed fusion. Appl. Surf. Sci..

[19] Harrison N.J., Todd I., Mumtaz K. (2015). Reduction of micro-cracking in nickel superalloys processed by Selective Laser Melting: A fundamental alloy design approach. Acta Mater..

[20] Raute J., Jokisch T., Biegler M., Rethmeier M. (2022). Effects on crack formation of additive manufactured Inconel 939 sheets during electron beam welding. Vacuum.

[21] Hong M., Wang S., Sun W., Geng Z., Xin J., Ke L. (2022). Effect of welding speed on microstructure and mechanical properties of selective laser melting Inconel 625 alloy laser welded joint. J. Mater. Res. Technol..

[22] Jokisch T., Marko A., Gook S., Üstündag Ö., Gumenyuk A., Rethmeier M. (2019). Laser Welding of SLM-Manufactured Tubes Made of IN625 and IN718. Materials.

[23] Brunner-Schwer C., Simón-Muzás J., Biegler M., Hilgenberg K., Rethmeier M. (2022). Laser welding of L-PBF AM components out of Inconel 718. Procedia CIRP.

[24] Li S., Hu Q.-W., Zeng X.-Y., Ji S.-Q. (2005). Effect of carbon content on the microstructure and the cracking susceptibility of Fe-based laser-clad layer. Appl. Surf. Sci..

[25] Zhang Z., Han Q., Liu Z., Wang L., Zhang H., Zhao P., Zhu G., Huang C., Setchi R. (2023). Cracking behaviour and its suppression mechanisms with TiB2 additions in the laser additive manufacturing of solid-solution-strengthened Ni-based alloys. Compos. Part B Eng..

[26] Hu P., Liu Z., Chen M., Li Y., Qi X., Xie J. (2024). Reducing cracking sensitivity of CM247LC processed via laser powder bed fusion through composition modification. J. Mater. Res. Technol..

[27] Caron J.L., Sowards J.W. (2014). Weldability of Nickel-Base Alloys. Compr. Mater. Process..

[28] Lin D., Xi X., Yan M., Ma R., Shi Z., Tang Z., Li Z., Tan C., Dong Z., Song X. (2023). Significantly improved weldability in laser welding of additively manufactured haynes 230 superalloys by tailoring microstructure. J. Mater. Res. Technol..

[29] Klarstrom D.L., Hoback G.L., Ishwar V.R., Qureshi J.I. (2000). Rejuvenation Heat Treatment and Weld Repairability Studies of HAYNES^®^ 230^®^ Alloy. Volume 4: Manufacturing Materials and Metallurgy; Ceramics; Structures and Dynamics; Controls, Diagnostics and Instrumentation; Education.

[30] Tomus D., Rometsch P.A., Heilmaier M., Wu X. (2017). Effect of minor alloying elements on crack-formation characteristics of Hastelloy-X manufactured by selective laser melting. Addit. Manuf..

[31] Zhou W., Fu W., Wang Y., Gao Z., Hu S., Song X., Kim H.S., Feng J. (2026). Semi-solid brazing via CoCrFeNiCux high-entropy alloy fillers: Interfacial microstructure, mechanical properties, and joining mechanism of C/C-SiC composites and TZM alloy. Adv. Compos. Hybrid Mater..

[32] Wei B., Liu Z., Cao B., Nong B., Zhang Y., Zhou H., Ai Y. (2022). Selective laser melting of crack-free René 104 superalloy by Sc microalloying. J. Alloys Compd..

[33] Zhao Y., Ma Z., Yu L., Liu Y. (2023). New alloy design approach to inhibiting hot cracking in laser additive manufactured nickel-based superalloys. Acta Mater..

[34] Yu Z., Guo C., Han S., Hu X., Cao L., Xu Z., Ding H., Zhu Q. (2021). The effect of Hf on solidification cracking inhibition of IN738LC processed by Selective Laser Melting. Mater. Sci. Eng. A.

[35] Griffiths S., Ghasemi Tabasi H., Ivas T., Maeder X., De Luca A., Zweiacker K., Wróbel R., Jhabvala J., Logé R.E., Leinenbach C. (2020). Combining alloy and process modification for micro-crack mitigation in an additively manufactured Ni-base superalloy. Addit. Manuf..

[36] Hu Y., Yang X., Kang W., Ding Y., Xu J., Zhang H. (2021). Effect of Zr content on crack formation and mechanical properties of IN738LC processed by selective laser melting. Trans. Nonferrous Met. Soc. China.

[37] Wang P., Shi W., Gu L., Ran H., Pan Z., Su Y., Song X., Cao J., Chen H., Li W. (2026). Exceptional mechanical properties of dual-phase heterogenous structure in Cf/C and superalloy joints via cold spray additive manufacturing-assisted brazing. J. Mater. Sci. Technol..

[38] Wang P., Liu W., Gu L., Ran H., Song X., Pan Z., Chen H., Li W. (2025). Integrated filler metal/base metal manufacturing via cold spray additive manufacturing for brazing Cf/SiC and superalloy. Trans. Mater. Res..

[39] Xi X., Lin D., He Z., Ma R., Wei H., Shi Z., Zhao W., Chen B., Tan C., Dong Z. (2025). Additively manufactured crack-free nickel-based superalloy with synergistic strength and plasticity via grain refinement and striped oxides inhibition. Compos. Part B Eng..

[40] Li R., Ma H., Wang R., Song H., Zhou X., Wang L., Zhang H., Zeng K., Xia C. (2025). Application of unsupervised learning methods based on video data for real-time anomaly detection in wire arc additive manufacturing. J. Manuf. Process..

[41] Li W., Zhang H., Wang G., Xiong G., Zhao M., Li G., Li R. (2023). Deep learning based online metallic surface defect detection method for wire and arc additive manufacturing. Robot. Comput.-Integr. Manuf..

[42] Bulatov V.V., Reed B.W., Kumar M. (2014). Grain boundary energy function for fcc metals. Acta Mater..

[43] Guo B., Zhang Y., Yang Z., Cui D., He F., Li J., Wang Z., Lin X., Wang J. (2022). Cracking mechanism of Hastelloy X superalloy during directed energy deposition additive manufacturing. Addit. Manuf..

[44] Li R., Ma H., Zeng K., Suo H., Li C., Fu Y., Zhang M., Zhang M., Fang X. (2025). Differential Evolution-Optimized Multi-Output Support Vector Regression-Based Prediction of Weld Bead Morphology in Wire-Fed Laser-Arc Directed Energy Deposition of 2319 Aluminum Alloy. Addit. Manuf. Front..

[45] Lin D., Hu J., Liu M., Li Z., Xi X., Dai J., Ma R., Shi Z., Tan C., Li R. (2024). Enhancing plasticity in laser additive manufactured high-entropy alloys: The combined effect of thermal cycle induced dissolution and twinning. Addit. Manuf..

[46] Burden M.H., Hunt J.D. (1974). Cellular and dendritic growth. II. J. Cryst. Growth.

[47] Mago J., Bansal S., Gupta D., Jain V. (2021). Influence of microwave heating on metallurgical and mechanical properties of Ni-40Cr3 C2 composite clads in the context of cavitation erosion resistance characteristics. Proc. Inst. Mech. Eng. Part C J. Mech. Eng. Sci..

[48] Zheng L., Li F., Zhou Y. (2012). Preparation, Microstructure, and Mechanical Properties of TiB_2_ Using Ti_3_AlC_2_ as a Sintering Aid. J. Am. Ceram. Soc..

[49] Savage W.F., Lundin C.F. (1965). The Varestraint test. Weld. J..

[50] Tan C., Li R., Su J., Du D., Du Y., Attard B., Chew Y., Zhang H., Lavernia E.J., Fautrelle Y. (2023). Review on field assisted metal additive manufacturing. Int. J. Mach. Tools Manuf..

[51] Wang P., Chen H., Pan Z., Shao H., Xiang J., Zhao S., Wang P., Li W. (2026). Electric field suppresses intermetallics via enhanced diffusion and non-equilibrium phase transformation solid solution in ultrafast brazed Ti_2_AlNb/Ti60 joints. J. Mater. Sci. Technol..

[52] Jiao Y., Huo Y., Fu W., Qi Y., Li X., Dong Y., Mei H., Song X. (2026). Microstructural evolution and mechanical properties of B_4_C-TiB_2_/GH99 joint brazed with AgCuTi filler. J. Eur. Ceram. Soc..

[53] Qi H., Zhou X., Li J., Hu Y., Xu L. (2021). Performance Testing and Rapid Solidification Behavior of Stainless Steel Powders Prepared by Gas Atomization. Materials.

[54] Aziz M.J. (1982). Model for solute redistribution during rapid solidification. J. Appl. Phys..

[55] Scheil E. (1942). Bemerkungen zur schichtkristallbildung. Int. J. Mater. Res..

[56] Swalin R.A., Martin A. (1956). Solute Diffusion in Nickel-Base Substitutional Solid Solutions. JOM.

[57] Després A., Antonov S., Mayer C., Tassin C., Veron M., Blandin J.-J., Kontis P., Martin G. (2021). On the role of boron, carbon and zirconium on hot cracking and creep resistance of an additively manufactured polycrystalline superalloy. Materialia.

[58] Lin D., Hu J., Wu R., Liu Y., Li X., SaGong M.J., Tan C., Song X., Kim H.S. (2024). Multiscale plastic deformation in additively manufactured FeCoCrNiMox high-entropy alloys to achieve strength–ductility synergy at elevated temperatures. Int. J. Plast..

[59] Hargather C.Z., Shang S.-L., Liu Z.-K. (2018). Data set for diffusion coefficients and relative creep rate ratios of 26 dilute Ni-X alloy systems from first-principles calculations. Data Brief..

[60] Hargather C.Z., Shang S.-L., Liu Z.-K. (2018). A comprehensive first-principles study of solute elements in dilute Ni alloys: Diffusion coefficients and their implications to tailor creep rate. Acta Mater..

[61] Laxmanan V. (1985). Dendritic solidification—I. Analysis of current theories and models. Acta Metall..

[62] Campbell C.E., Boettinger W.J., Kattner U.R. (2002). Development of a diffusion mobility database for Ni-base superalloys. Acta Mater..

[63] Rappaz M., Boettinger W.J. (1999). On dendritic solidification of multicomponent alloys with unequal liquid diffusion coefficients. Acta Mater..

