# Supplementary material for: Weldability Improvement of Additively Manufactured Nickel-Based Superalloy by Controlling W and Si Migration at Solidification Front
Source: Materials (Basel). 2026 Jul 27;19(15):3210. doi: 10.3390/ma19153210 (PMC13466282; doi:10.3390/ma19153210)
Supplement: Supplementary file 1 [file materials-19-03210-s001.zip › materials-4391119-supplementary.pdf]

According to the theoretically calculated solidification path, Figure S1 illustrates a schematic diagram of solute element migration during solidification. Due to the high solidification temperature, a Ni-based solid solution with a high melting point is preferentially generated in the early stages of solidification. The strong convection induced by the laser action resulted in a relatively homogeneous distribution of elements in the liquid phase. As the solidification process proceeded, the substructure growth impeded the melt flow along the solidification front, and the degree of convection caused by the laser action in the inter-substructures gradually decayed. The enrichment of solute elements gradually occurred at the solidification front, resulting in progressively severe solute trapping. The concentration of solute elements in the solid phase increased, and the reverse diffusion driving force improved; the concentrations of solute elements in the solid and liquid phases at the solidification front were similar. In the late and final stages of solidification ($0.8 < f_s < 1.0$), the C and Cr contents in the liquid phase decreased successively because of the first precipitation of $M_{23}C_6$. In contrast, the decrease in W content was probably due to the precipitation of the TCP phase and silicides, which corresponded well with Figures 5 and 6. However, because of the small diffusion coefficient of W in the solid phase, very few atoms entered the liquid phase via reverse diffusion after the generation of the trace TCP phase and silicides at the end of solidification. The variation trend of the Si-content difference in the liquid phase was reversed from that in the solid phase, and the variation inflection points were consistent, further supporting the final precipitation of the Si-rich phase. The B and C atoms have a higher diffusion rate and can diffuse between the solid and liquid phases more easily, leading to a sharp increase in the content of C and B in the final solidified solid phase. The enlarged low-melting-point mushy zone at the GB inclined the remaining liquid metal to produce low-melting-point $M_x(B, C)_y$ phases rather than carbides, which extended the solidification cracking tendency.

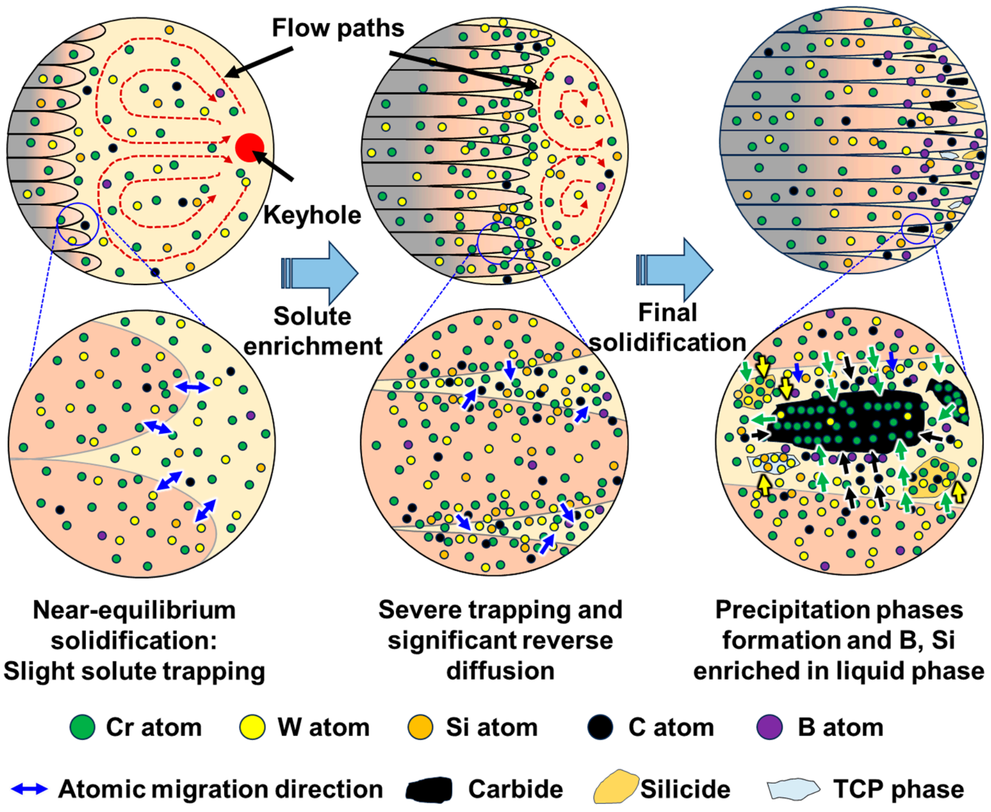

Figure S1. Schematic diagram of solute element migration during solidification.
